# Risk Factors for Radiographic Tibiofemoral Knee Osteoarthritis: The Wuchuan Osteoarthritis Study

**DOI:** 10.1155/2010/385826

**Published:** 2010-12-28

**Authors:** Jianhao Lin, Rujun Li, Xiaozheng Kang, Hu Li

**Affiliations:** ^1^Arthritis Institute, Peking University, People's Hospital, Beijing 100044, China; ^2^Department of Thoracic Surgery, Beijing Cancer Hospital and Institute, Beijing 100142, China

## Abstract

*Objective*. To investigate the risk factors of radiographic tibiafemoral knee osteoarthritis (OA). *Methods*. A population-based cross-sectional survey was conducted in Wuchuan County. A questionnaire and bilateral weight-bearing posterior-anterior semi-flexed knee radiographs were completed and read for Kellgren and Lawrence (K/L) grade and joint space narrowing (JSN; 0–3 scale) in each compartment. An logistic regression analysis was performed for radiographic tibiafemoral, lateral compartment, and medial compartment knee OA, respectively. Adjusted odds ratios (OR) and 95% confidence intervals (CI) were calculated. *Results*. Age, sex, and knee injury were strongly associated with tibiafemoral, lateral and medial compartment knee OA. BMI also had a dose-response relationship with them. Physical activity level, and physical activity exposure at work, not significantly though, were associated with an elevated risk for this three kinds of knee OA. *Conclusions*. Physical activity exposure increased the risk of knee OA. It was likely to be the heavier physical activity in Wuchuan osteoarthritis study that counteracted the BMI gap compared with the Beijing and the Framingham OA study. We verified that Chinese had a more valgus alignment of the knee compared with Caucasian population, and this provide a possible explanation why Chinese have a higher prevalence of lateral compartment OA.

## 1. Introduction

 Knee osteoarthritis (OA) is an important cause of pain and disability in old population, and it is becoming increasingly prevalent worldwide due to its association with an aging population and due to a growing prevalence of obesity [1]. To investigate the prevalence and risk factors of knee osteoarthritis, many population-based observational studies have been conducted worldwide. The etiology of knee OA is believed to be multifactorial, and the following risk factors have been identified: heredity [2–4], obesity [5–8], injury [8–11], and physical workload [12–15]. However, most of the large-scale studies were conducted in North America and Europe, with scant information from less developed regions Reference [16], and most of the study participants were based on urban residents or workers, while people in rural areas, especially farmers, were rarely investigated. In fact, farmers form a large proportion of the population in less developed regions, most of whom have to endure heavy physical workload until an old age. The prevalence and risk factors of knee OA among these people might be different. 

 We conducted a population-based cross-sectional survey in Wuchuan county, a remote rural region in Inner Mongolia of northern China. Most of the participants were farmers reporting heavy physical occupational activity. We found that the prevalence of severe radiographic and symptomatic knee OA was much higher than that reported from urban regions of China or in the Framingham cohort [16]. We estimates the risk of knee osteoarthritis related to the following factors: age, sex, BMI, knee injury, physical activity level, and physical activity exposure at work including walking, walking roughly, cycling, standing, digging, kneeling, squatting, climbing, and lifting of loads. To allow valid comparisons with the Beijing OA study and the Framingham cohort, the same survey instruments and comparable radiographic protocols were used.

## 2. Subjects and Methods

### 2.1. Study Population

The study design has been described in detail in a previous publication [16]. Briefly, A total of 1165 individuals reporting to be aged 50 years and older were identified in 762 randomly selected households in Wuchuan County, Inner Mongolia. Of these, 27 subjects were excluded from further study participation. Of eligible participants, 90% completed the home interview and attended the radiographic examination, and these subjects were included in the analysis.

A total of 1025 participants (520 women and 505 men) were involved in the analysis, over 90% living on farming as reported. The survey questionnaire focusing on joint symptoms and possible risk factors for knee OA was completed under the supervision of the intensively trained interviewers who went door to door to enumerate and interview all men and women. A posterior-anterior weight-bearing semi-flexed view radiographs were taken of both knees, strictly according to a validated acquisition protocol [17]. Radiographs were read by the chief investigator XK using the OARSI atlas, and Kellgren-Lawrence grades (range 0–4) were also assigned. Radiographic knee OA was defined as having a Kellgren-Lawrence grade ≥2 in one or both knees. Similarly, severe radiographic knee OA was defined as having a Kellgren-Lawrence grade ≥3 in one or both knees. We defined medial and lateral radiographic OA, respectively, if a knee had a Kellgren-Lawrence grade ≥2 and medial or lateral joint space narrowing score ≥1.

### 2.2. Statistical Analysis

Logistic regression analysis for the parameters sex, age, BMI, knee injury, physical activity level, and physical activity exposure at work including walking, walking roughly, cycling, standing, digging, kneeling, squatting, climbing, and lifting of loads was performed for radiographic tibiafemoral knee OA, lateral compartment knee OA, and medial compartment knee OA, respectively. Parameters that have significant influence were included in the regression equations, and odds ratios (OR) and 95% confidence intervals (CI) for these parameters were calculated. Then we calculated the adjusted OR (adjusted for the parameters included in the equations) and 95% CI for the rest of parameters which were excluded in the regression equations. For better analysis, the parameter age was divided into four grades: 50 to 52.5, >52.5 to <60, 60 to <70, and ≥70 years old, and BMI was divided into four grades, too: <18.5, ≥18.5 to <25.0, ≥25.0 to <28.0, and ≥28.0 kg/m^2^.

## 3. Results

The characteristics of the participants are presented in Table 1. As described in the previous publication. At the time of data analysis, it was discovered that 24 men and 26 women were actually younger than 50 years (either 48 or 49 years). We retained these 50 participants in the 50–52.5 years of age category. Almost all participants (91%) were farmers or had been engaged in farming as their main occupation, with the remaining 9% reporting their main occupation as businessman or shopkeeper. At the time of the survey, 85% of all participants were still working. Most of participants (91%) reported that the occupation they had held the longest involved heavy physical work. The prevalence rates of radiographic tibiofemoral knee OA, lateral compartment knee OA, and medial compartment knee OA are presented in Table 2.

## 4. Risk Factors for Radiographic Tibiofemoral Knee OA

Age, sex, BMI, and knee injury were included in the regression equation for the radiographic tibiofemoral knee OA risks. A steep dose-response relationship between age and the diagnosis of knee osteoarthritis was found: compared with persons at least 70 years old (group 4), persons about 50 years old (group 1) had a much lower knee osteoarthritis risk (OR 0.065; 95% CI 0.034–0.123: Table 3). The mean BMI was also strongly associated with knee osteoarthritis: compared to persons with a BMI at least 28.0 kg/m^2^, persons with a BMI of less than 18.0 kg/m^2^ only had an OR of 0.15 (95% CI 0.06–0.38). Assuming a linear dose-response relation, the risk would halve for each 4.2 kg/m^2^ decrease in the BMI. Males had an OR of 0.45 compared with females; this means females have more than a 2-fold radiographic tibiofemoral knee OA risk than males. Participants without knee injury showed an OR of 0.43 (95% CI 0.23–0.81) for the risk of knee osteoarthritis, in comparison to persons with knee injury. 

We calculated the adjusted ORs for the parameters not in the regression equation (adjusted for age, sex, BMI, and knee injury) (Table 3). Compared with heavy physical level, participants with moderate physical level had a lower adjusted OR (0.81, 95% CI 0.44–1.48). Persons cycling or digging less than 2 hours per day had a lower radiographic tibiofemoral knee OA risk in comparison to persons cycling or digging at least 2 hours per day (OR 0.71 or 0.72, 95% CI 0.44–1.16 or 0.48–1.08), indicating that cycling and digging increase the risk of radiographic tibiofemoral knee OA despite that the influence was not statistically significant (*P* > .05). Kneeling elevated the risk slightly with an adjusted OR of 0.93 (95% CI 0.64–1.35) for kneeling less than 30 minutes per day compared with at least 30 minutes per day. However, lifting or moving objects weighing 10 kilograms or more did not increase the radiographic tibiofemoral knee OA risk with an adjusted OR of 0.99 (95% CI 0.55–1.80), while squatting even decreased the risk of radiographic tibiofemoral knee OA with an adjusted OR of 1.25 (95% CI 0.85–1.84) for kneeling at least 30 minutes per day compared with squatting at least 30 minutes per day.

## 5. Risk Factors for Lateral Compartment Knee OA

Age, sex, knee injury, and squatting were included in the regression equation for the lateral compartment knee OA risk (Table 3). A dose-response relationship between age and the lateral compartment knee OA was found, too, although the correlation was not as significant as that of age and radiographic tibiofemoral knee OA. Compared with radiographic tibiofemoral knee OA, Similar results were found for sex and knee injury in influencing the risk of the lateral compartment knee OA. Unexpectedly, squatting decreased the lateral compartment knee OA risk with an adjusted OR of 2.67 (95% CI 1.50–4.76) for kneeling at least 30 minutes per day compared with squatting at least 30 minutes per day, and it was statistically significant (*P* < .05). No significant effect on the lateral compartment knee OA risk was found for BMI. 

We calculated the adjusted ORs for the parameters not in the regression equation (adjusted for age, sex, BMI, and squatting) (Table 3). Participants digging less than 2 hours per day had a lower lateral compartment knee OA risk in comparison to the ones digging at least 2 hours per day (OR 0.71 or 0.72, 95% CI 0.44–1.16 or 0.48–1.08); this means that digging increases the lateral compartment knee OA risk. So did lifting and kneeling. As showed in Table 3, subjects who did not lift or move objects weighing 10 kilograms or more had an adjusted OR of 0.76 (95% CI 0.31–1.86), and the adjusted OR for kneeling less than 30 minutes per day was 0.83 (95% CI 0.47–1.47) compared with at least 30 minutes per day. Cycling elevated the risk slightly with an adjusted OR of 0.93 (95% CI 0.43–2.01) for cycling less than 2 hours per day compared with at least 2 hours per day. BMI did not show a definite trend for the risk of lateral compartment knee OA. Compared with BMI ≥ 28.0 kg/m^2^, the adjusted ORs of BMI < 18.5, ≥18.5 to <25.0, and ≥25.0 to <28.0 kg/m^2^ were 1.69 (0.37–7.59), 1.09 (0.31–3.81), and 2.36 (0.62–8.95), respectively. However, we should note that the number of participants with lateral compartment knee OA in BMI < 18.5 kg/m^2^group and BMI ≥28.0 kg/m^2^ group were too small, only 6 and 3, respectively, to make the analysis result inconvincible. A heavier physical work level did not lead to a higher risk of lateral compartment knee OA, though, with an adjusted OR 1.30 (95% CI 0.57–2.99) for moderate physical work compared with heavy physical work.

## 6. Risk Factors for Medial Compartment Knee OA

Age, sex, and BMI were included in the regression equation for the medial compartment knee OA risk (Table 3). A dose-response relationship between age, BMI, and the medial compartment knee OA was found too, similar with radiographic tibiofemoral knee OA. Compared with persons at least 70 years old (group 4), persons at 50 to 52.5, >52.5 to <60, and 60 to <70 years old had a lower medial compartment knee OA risk with ORs (95% CI) of 0.037 (0.017–0.082), 0.089 (0.047–0.17), and 0.39 (0.22–0.70), respectively. The mean BMI was also strongly associated with medial compartment knee OA Compared to persons with a BMI ≥ 28.0 kg/m^2^, persons with a BMI < 18.5, ≥18.5 to <25.0, and ≥25.0 to <28.0 kg/m^2^ had ORs (95% CI) of 0.13 (0.043–0.36), 0.20 (0.095–0.42), and 0.44 (0.19–1.00), respectively. Females had a higher medial compartment knee OA risk: the risk of females was 1.5-fold higher than that of males.

The adjusted ORs for the parameters not in the regression equation (adjusted for age, sex, and BMI) were calculated as showed in Table 3. The medial compartment knee OA risk of participants with knee injury was almost double that of participants without knee injury (adjusted OR (95% CI): 0.52 (0.24–1.10)), and it was statistically significant (*P* = .085). Heavier physical workload, cycling, and digging lead to a higher medial compartment knee OA risk. Lifting or moving objects weighing 10 kg or more also increased the medial compartment knee OA risk slightly. Persons kneeling and squatting did not show a higher medial compartment knee OA risk, though. Squatting even decreased the risk, with the adjusted OR (95% CI) of 1.15 (0.73–1.80) for kneeling less than 30 minutes per day compared with squatting at least 30 minutes per day.

## 7. Discussion

Our results showed that age, sex, and previous knee injury were strongly associated with radiographic tibiofemoral knee OA, lateral compartment knee OA, and medial compartment knee OA. Age had a steep dose-response relationship with the risk of all of the three kinds of knee OA. Females or participants with previous knee injury approximately had a 2-fold risk compared with males or those without. BMI had a definite dose-response relationship with the risk of radiographic tibiofemoral knee OA and medial compartment knee OA, while the relationship between BMI and lateral compartment knee OA had an inexact trend. However, we should note that the number of participants with lateral compartment knee OA in BMI < 18.5 kg/m^2^  group and BMI ≥ 28.0 kg/m^2^ group was too small, only 6 and 3 respectively, to make the analysis result inconvincible. Excluding these two groups, we found that persons of BMI ≥ 25.0 to <28.0 kg/m^2^ had more than a 2-fold risk of lateral compartment knee OA compared with persons of BMI ≥ 18.5 to <25.0 kg/m^2^, indicating that elevated BMI increased the risk of lateral compartment knee OA, too.

Heavier physical workload showed a positive association with radiographic tibiofemoral and medial compartment knee OA, but a negative association with lateral compartment knee OA. One explanation is that the number of participants with lateral compartment knee OA in moderate physical workload level group was small, only 9. In our study, no one reported that the physical activity level was sedentary; 7 persons reported it was light physical activity level, and we excluded this group for analysis; nearly 8% subjects (84 persons) reported it was moderate physical activity level, while more than 91% persons reported the physical activity level was heavy. Thus, the influence of physical workload on the risk of knee OA was hard to be highlighted, as most of the participants had heavy physical activity. This may explain the result that physical activity exposure such as cycling, digging, lifting or moving objects, and kneeling only had a slight or moderate effect on the risk of knee OA. Another explanation for this result is that we did not make detailed levels for these kinds of physical activity exposure, we just roughly classified them into two levels. For instance, digging was only divided into digging 2 hours or more per day or less than 2 hours. And we did not estimate the accumulative time of exposure. In this case, the influence of physical activity exposure was hard to stand out. In addition, squatting decreased the risk of radiographic tibiofemoral, lateral compartment, and medial compartment knee OA. This effect was even statistically significant for lateral compartment knee OA (*P* = .001). This result could be explained by the biomechanical role of loading on the pathogenesis of knee OA. During deep knee flexion, such as kneeling and squatting, sagittal movements were 2-3 times higher than they were during walking [17]. These high sagittal movements would be expected to increase loads across the patellofemoral joint. While repeated squatting and kneeling may well have consequences for the tibiofemoral joint, the part of the knee most likely to be exposed to the highest load during such activities is the patellofemoral joint. Amin et al. [18] found frequent squatting/kneeling lead to a greater likelihood for worse cartilage morphology scores at the patellofemoral joint, while had no significant influence on the tibiofemoral joint. However, other studies [19, 20] showed that squatting increased the risk of tibiofemoral knee OA, while the association with patellofemoral knee OA was weaker or doubtful.

As noted in the previous publication [16], the overall prevalence of radiographic knee OA in the compared age group was similar to that demonstrated in the Beijing OA, despite lower BMI (22-23 kg/m^2^ versus 25-26 kg/m^2^), which is a known risk factor for knee OA. The data on the correlation between physical activity level/exposure and knee OA would suggest that it was likely to be the heavier physical activity in Wuchuan osteoarthritis study that counteracted the BMI gap. But it is hard to confirm this hypothesis by internal comparison, as most of participants had a high physical activity level. However, although the comparison between these two cohorts was unavailable, this result reflected the effect of physical workload on the risk of knee OA.

Harvey et al. [21] measured knee alignment of participants without knee OA from the Beijing osteoarthritis study and the Framingham osteoarthritis study, respectively. They found that this there was a more valgus alignment in Chinese cohorts, and that possibly explains why the Chinese had a higher lateral compartment OA with lower prevalence of obesity compared with Caucasian communities. Using the similar methods as the Beijing osteoarthritis study and the Framingham osteoarthritis study, the Wuchuan osteoarthritis study showed a similar prevalence of lateral compartment disease with the Beijing osteoarthritis study, and it was two to three times higher in both Chinese cohorts compared with estimates from the Framingham OA Study. We measured the femoral tibial angle (FTA) of subjects in our study, and calculated the mean FTA (±SD) of knees (*N* = 1737) without radiographic OA. The knee alignment (FTA (±SD): 4.9 (±3.1)) of Wuchuan cohorts without OA was a little more valgus than the Beijing cohorts (anatomic axis angle: 4.32°), and more obviously valgus than the Framingham cohorts (anatomic axis angle: 2.59°). The more valgus alignment of the knee in the Chinese would serve to shift the mechanical loading towards the lateral compartment, and provide a possible explanation why the Chinese have a higher prevalence of lateral compartment OA.

There are several limitations in our study. Most of the participants were living on farming, and more than 90 percent persons had heavy physical work on the job. This made it hard to highlight the effects of physical activity level and exposure on the knee OA risk as the lack of lower physical activity population. Another is that we just investigated the rough hours of those physical activity exposure (e.g., digging <2 hours/day or ≥2 hours/day) instead of the exact number of hours, thus it is hard to perform correlation analysis of the activity and knee OA, which is better to reflect the relationship of physical activity exposure and knee OA. We also have to mention that the average age of the participants in the study was only 55 to 57 years old, which might have a lot to do with the relatively low overall prevalence rate of knee OA. In addition, our knee alignment (FTA) measures were taken manually with goniometer with 1 degree precision instead of measuring on full-length radiographs. The values of FTA and anatomic axis angle would be similar theoretically, but this similarity needs to be further verified and there might be an error of manual measurement with goniometer. Further this is a cross-sectional analysis and the relations between potential risk factors and knee OA need to be further examined in longitudinal samples. No firm conclusions can be drawn from this cross-sectional study.

## 8. Conclusions

We found that aging, female gender, obesity, and knee injury had a strong association with radiographic tibiofemofal knee OA risk, and similar association existed for medial or lateral compartment knee OA. Heavier physical activity increased the risk of radiographic tibiofemofal knee OA, lateral compartment knee OA, and medial compartment knee OA. Physical activity exposure such as cycling, kneeling, lifting, and digging had a light to moderate effect on the elevation of knee OA risk. Although this effect was not that obvious via internal comparison, it was likely to be the heavier physical activity in Wuchuan osteoarthritis study that counteracted the BMI gap compared with the Beijing and the Framingham OA studies. We verified that the Chinese had a more valgus alignment of the knee compared with Caucasian population, and this provides a possible explanation why Chinese have a higher prevalence of lateral compartment OA. Future work in this area should focus on the longitudinal study of these potential risk factors and their relationship to the development of OA.

## Figures and Tables

**Table 1:** Characteristics of the Wuchuan County study participants.

	Men	Women
No.	505	520
Age (years ± SD)	57.3 ± 8.3	55.6 ± 7.5
Weight (kg ± SD)	59.7 ± 8.9	56.1 ± 9.5
BMI (kg/m^2^ ± SD)	21.6 ± 2.7	23.3 ± 3.5
Main occupation farming (%)	94	88
Still working (%)	90	81

**Table 2:** Prevalence rates of radiographic tibiofemoral, lateral compartment, and medial compartment knee OA.

	Men	Women
No. of rTFKOA1 (PR4)	53 (10.5%)	102 (19.6%)
No. of LCKOA2 (PR)	18 (3.6%)	35 (6.7%)
No. of MCKOA3 (PR)	37 (7.3%)	71 (13.7%)

^1^Radiographic tibiofemoral knee OA.

^2^Lateral compartment knee OA.

^3^Medial compartment knee OA.

^4^Prevalence rate.

**Table 3:** Risk factors for the radiographic tibiofemoral, lateral compartment, and medial compartment knee OA.

Group		rTFKOA^1^			LCKOA^2^			MCKOA^3^		
		No. (%) of subjects	ORs* (95% CI)	*P* for trend	No. (%) of subjects	ORs (95% CI)	*P* for trend	No. (%) of subjects	ORs (95% CI)	*P* for trend
Age	1 (youngest)	24 (6.9)	0.065 (0.034–0.123)	<.001	11 (3.2)	0.28 (0.11–0.69)	.006	11 (3.2)	0.037 (0.017–0.082)	<.001
	2	37 (10.4)	0.12 (0.064–0.21)	<.001	12 (3.4)	0.32 (0.13–0.77)	.012	24 (6.7)	0.089 (0.047–0.17)	<.001
	3	53 (26.0)	0.50 (0.29–0.85)	.010	20 (9.8)	1.1 (0.49–2.5)	.818	38 (18.6)	0.39 (0.22–0.70)	.001
	4 (oldest)	41 (35.0)	1.0		10 (8.5)	1.0		35 (29.9)	1.0	
Sex	1 (male)	53 (10.5)	0.45 (0.30–0.67)	<.001	18 (3.6)	0.48 (0.26–0.88)	.017	37 (7.3)	0.40 (0.25–0.64)	<.001
	2 (female)	102 (19.6)	1.0		35 (6.7)	1.0		71 (13.7)	1.0	
BMI	1 (thinnest)	11 (14.5)	0.15 (0.06–0.38)	<.001	6 (7.9)	1.69 (0.37–7.59)	.497	8 (10.5)	0.13 (0.043–0.36)	<.001
	2	91 (12.2)	0.21 (0.11–0.40)	<.001	32 (4.3)	1.09 (0.31–3.81)	.894	66 (8.9)	0.20 (0.095–0.42)	<.001
	3	32 (23.2)	0.60 (0.29–1.21)	.153	12 (8.7)	2.36 (0.62–8.95)	.208	19 (13.8)	44 (0.19–1.00)	.051
	4 (fattest)	21 (31.8)	1.0		3 (4.5)	1.0		15 (22.7)	1.0	
Injury	0 (no)	137 (14.4)	0.43 (0.23–0.81)	.009	45 (4.7)	0.31 (0.13–0.71)	.006	97 (10.2)	0.52 (0.24–1.10)	.085
	1 (yes)	17 (23.6)	1.0		8 (11.1)	1.0		11 (15.3)	1.0	
Workload^4^	3 (moderate)	20 (23.8)	0.81 (0.44–1.48)	.487	9 (10.7)	1.30 (0.57–2.99)	.536	14 (16.7)	0.66 (0.33–1.32)	.240
	4 (heavy)	134 (14.3)	1.0		43 (4.6)	1.0		93 (10.0)	1.0	
Driving	<2 h/d	149 (16.3)	1.12 (0.44–2.83)	.809	50 (5.5)	0.89 (0.24–3.25)	.861	104 (11.4)	0.94 (0.31–2.87)	.912
	≥2 h/d	6 (5.3)	1.0		3 (2.7)	1.0		4 (3.5)	1.0	
Cycling	<2 h/d	127 (15.6)	0.71 (0.44–1.16)	.175	44 (5.4)	0.93 (0.43–2.01)	.844	90 (11.0)	0.75 (0.42–1.34)	.33
	≥2 h/d	28 (13.4)	1.0		9 (4.3)	1.0		18 (8.6)	1.0	
Digging	<2 h/d	102 (14.3)	0.72 (0.48–1.08)	.109	35 (4.9)	0.65 (0.35–1.21)	.178	74 (10.3)	0.86 (0.54–1.38)	.532
	≥2 h/d	53 (17.1)	1.0		18 (5.8)	1.0		34 (11.0)	1.0	
Kneeling	<0.5 h/d	78 (14.3)	0.93 (0.64–1.35)	.690	24 (4.4)	0.83 (0.47–1.47)	.529	56 (10.3)	0.99 (0.64–1.53)	.962
	≥0.5 h/d	77 (16.0)	1.0		29 (6.0)	1.0		52 (10.8)	1.0	
Squatting	<0.5 h/d	58 (17.4)	1.25 (0.85–1.84)	.267	28 (8.4)	2.67 (1.50–4.76)	.001	40 (12.0)	1.15 (0.73–1.80)	.545
	≥0.5 h/d	97 (14.0)	1.0		25 (3.6)	1.0		68 (9.8)	1.0	
Lifting^5^	0 (no)	20 (23.0)	0.99 (0.55–1.80)	.976	8 (9.2)	0.76 (0.31–1.86)	.549	15 (17.2)	0.94 (0.49–1.81)	.849
	1 (yes)	135 (14.4)	1.0		45 (4.8)	1.0		93 (9.9)	1.0	

*Oddsratios (ORs) were adjusted for age, sex, BMI, and knee injury.

^1^Radiographic tibiofemoral, lateral compartment, and medial compartment knee OA.

^2^Lateral compartment knee OA.

^3^Medial compartment knee OA.

^4^The subgroup “2” representing light physical activity was not included in analysis for its number of subjects was too small (only 8).

^5^Lift or move objects weighing 10 kilograms or more almost everyday.
